# Expression of Somatostatin Receptor 2 in Somatotropinoma Correlated with the Short-Term Efficacy of Somatostatin Analogues

**DOI:** 10.1155/2017/9606985

**Published:** 2017-03-15

**Authors:** Wenjuan Liu, Lina Xie, Min He, Ming Shen, Jingjing Zhu, Yeping Yang, Meng Wang, Ji Hu, Hongying Ye, Yiming Li, Yao Zhao, Zhaoyun Zhang

**Affiliations:** ^1^Division of Endocrinology and Metabolism, Huashan Hospital, Fudan University, Shanghai 200040, China; ^2^Institute of Endocrinology and Diabetology, Fudan University, Shanghai 200040, China; ^3^Department of Endocrinology, Kunshan Rehabilitation Hospital, Suzhou, Jiangsu 215314, China; ^4^Department of Neurosurgery, Huashan Hospital, Fudan University, Shanghai 200040, China; ^5^Shanghai Pituitary Tumor Center, Shanghai 200040, China; ^6^Department of Pathology, Huashan Hospital, Fudan University, Shanghai 200040, China; ^7^Department of Endocrinology, The Second Affiliated Hospital, Soochow University, Suzhou, Jiangsu 215004, China

## Abstract

The expression of somatostatin receptor subtypes (SSTRs) in pituitary growth hormone- (GH-) secreting adenomas may predict the response to somatostatin analogues (SSA). Our aim was to evaluate the value of the immunohistochemical (IHC) scores of 2 subtypes, SSTR2 and SSTR5, in predicting the short-term efficacy of SSA therapy in patients with active acromegaly. Ninety-three newly diagnosed acromegalic patients were included in our study. These patients were categorized into either a SSA-pretreated group (SA, *n* = 63) or a direct-surgery group (DS, *n* = 30), depending on whether or not presurgical SSA treatment was received. IHC analysis, using a 12-grade scoring system, with rabbit monoclonal antibodies against SSTR2 and SSTR5, was performed on all adenoma tissues. The reduction of GH, IGF-1, and tumor size after treatment with SSA for 3 months was measured. Compared with that in the DS group, SSTR2 expression was lower in the SA group. Additionally, in the SA group, SSTR2 expression was positively correlated with the reduction of IGF-1 and tumor volume. However, there was no correlation between the SSTR5 score and the efficacy of SSA. In conclusion, the protein expression of SSTR2, but not of SSTR5, is a valuable indicator in predicting biochemical and tumor size response to short-term SSA treatment in acromegalic patients.

## 1. Introduction

Somatostatin analogues (SSA) are the most widely used medications for the treatment of active acromegaly [1]. They exert biological effects by binding to G protein-coupled receptors known as somatostatin receptors (SSTR), mainly SSTR2 and SSTR5. The overall efficacy of SSA is ~35% in biochemical remission rate (mean GH levels < 2.5 *μ*g/L and IGF-1 normalization) [2, 3] and ~70% in oncological response rate (tumor volume shrinkage > 20%) [4, 5]. Therefore, it is advantageous to identify patients who may respond to SSA.

Clinical and experimental studies have thus far been focused on predictors related to SSA response such as age, gender, GH levels, T2-weighted signal intensity on MRI, indium-111 octreotide scanning, and SSTR subtype expression patterns [2, 3, 5–10]. However, different studies have shown conflicting results which cannot be translated directly to clinical practice [5, 11, 12].

The predictive value of SSTR expression is still a matter of controversy. Previous studies have been focused on SSTR2 and SSTR5, as octreotide binds to these two receptors with high affinity [13]. However, only SSTR2 was reported to be associated with the acute and long-term effects of octreotide in most studies [9, 14, 15]. In addition, data about the predictive value of SSTR in the Chinese population are lacking, and the distribution patterns of SSTR in normal pituitary and somatotrophic adenomas have not been reported. In this study, we examined the value of SSTR expression in predicting the short-term efficacy of SSA and the distribution patterns of SSTR in the normal pituitary and somatotrophic adenomas.

## 2. Materials and Methods

### 2.1. Patients and Samples

Normal pituitary samples with no clinical or pathological evidence of endocrine disorders (*n* = 20, normal pituitary group, NP group) were obtained via autopsy. Pathological examination excluded the presence of pituitary adenomas. We recruited 97 newly diagnosed and untreated patients with acromegaly at a tertiary referral center in East China from September 2008 to August 2013. Four patients were excluded from our study because of sparse adenoma tissue in the remaining specimens. Acromegaly was diagnosed according to clinical features including failure of GH suppression to below 1 *μ*g/L in response to a 75 g oral glucose load, serum insulin-like growth factor 1 (IGF-1) levels above the age-matched reference range, and radiological evidence of pituitary tumors. Enhanced MRI was performed to identify the size and position of the pituitary adenomas. 93 patients were included in the statistical analysis with baseline clinical characteristics presented in Table 1. For these patients, the decision regarding primary treatment was made based on guidelines for acromegaly, clinical evaluation by a multidisciplinary pituitary tumor conference (consisting of endocrinologists, neurosurgeons, radiologists, and radiation therapists), and the patients' preference.

Of the 93 patients, 63 were pretreated with long-acting SSA (octreotide LAR or lanreotide SR, SA group) for 3 months prior to their surgeries, and the other 30 underwent surgery without medical pretreatment (direct surgery, DS group). Patients were evaluated after 3 months of SSA treatment. Random GH, IGF-1, and pituitary MRI were performed to evaluate the response to SSA. Biochemical response was defined as a posttreatment random GH on GH day curve < 2.5 *μ*g/L or >75% fall compared with the pretreatment random GH [16]. Oncological response was defined as tumor volume shrinkage > 20% [17].

The Institutional Review Board at Huashan Hospital approved this study, and written informed consent was obtained from all patients before study entry.

### 2.2. Biochemical Measurements

IGF-1 was measured with Immulite 2000 solid-phase, which is an enzyme-labeled chemiluminescent immunometric assay (Siemens Healthcare Diagnostic Products Limited, UK). The normal ranges were age-dependent (1–6 years: 49–327 *μ*g/L; 7–11 years: 57–551 *μ*g/L; 12-13 years: 143–850 *μ*g/L; 14–16 years: 220–996 *μ*g/L; 17-18 years: 163–731 *μ*g/L; 19-20 years: 127–483 *μ*g/L; 21–35 years: 115-358 *μ*g/L; 36–50 years: 94–284 *μ*g/L; and >50 years: 55–238 *μ*g/L) [18]. The IGF-1 index, a parameter that describes the level of the IGF-1 based on age (defined as the ratio of IGF-1 value to the maximum of reference ranges), was calculated for all patients. GH was measured by a two-site chemiluminescent immunometric assay using AutoDELFIA® hGH (PerkinElmer Life and Analytical Sciences, Wallac Oy).

### 2.3. Immunohistochemistry

The pituitary glands were fixed in 4% paraformaldehyde overnight and then embedded in paraffin. The sections (5 microns) were deparaffinized in methanol for 15 minutes and then treated with 10 mM sodium citrate for 1 hour to unmask the antigen epitopes. Following this, endogenous peroxidase activity was blocked by treatment with 3% hydrogen peroxide for 30 minutes. Rabbit monoclonal antibodies for SSTR2 (1 : 100, ab134152, Abcam, US) and SSTR5 (1 : 100, ab109495, Abcam, US) were then examined as previously described [15]. The adenomas were scored using the immunoreactive score (IRS) performed by two researchers who were blinded regarding the clinical data. The IRS (0–12) is the product of the proportion of immunoreactive cells (0, 0%; 1, <10%; 2, 10%–50%; 3, 51%–80%; or 4, >80%) and the staining intensity (0, no staining; 1, weak; 2, moderate; and 3, strong). 10 pictures of each slice were analyzed, and the mean IRS was obtained for the following analysis.

### 2.4. Statistical Analysis

Data were presented as mean ± (or median with interquartile range) for continuous variables that were normally (or not normally) distributed and as frequencies for categorical variables. The normal distribution of continuous parameters was analyzed by the Shapiro-Wilk test. After log transformation, the baseline GH concentration, IGF-1 index, and tumor volume were normally distributed. Student's *t*-test was used to analyze 2 groups of normally distributed continuous variables before or after log transformation. The significance of the differences in mean values among different groups was evaluated using one-way ANOVA followed by the Tukey test. To analyze the correlations between the IRS and clinical parameters, the Pearson correlation coefficient or the Spearman rank correlation coefficient was calculated when variables were normally or not normally distributed, respectively. Following this, parametric or nonparametric significance testing was performed. Statistical analyses were performed using the statistical package SPSS for Mac Ver. 20.0 (SPSS Inc., Chicago, IL, USA). *P* values less than 0.05 were considered to be statistically significant.

## 3. Results

### 3.1. Basic Characteristics of the Cohort and the Differences between the SA and DS Groups

Table 1 shows the clinical information of the 93 patients, of which 59 are female and 34 are male. 63 patients received 3-month presurgical SSA treatment (SA group), and the other 30 patients underwent surgery directly without any pretreatment (DS group). The average age at diagnosis was 42 y (IQR 35–53 y). The mean baseline growth hormone level was 34.2 *μ*g/L (IQR 13.5–57.9 *μ*g/L), while the mean IGF-1 index was 2.9 (IQR 2.4–3.6). Data for tumor volumes were available in 77 cases, and the mean baseline tumor volume was 1900 mm^3^ (IQR 1000–2515 mm^3^).

Regarding the baseline characteristics of these two groups, data in Table 1 also showed that the IGF-1 index was higher in the SA group (3.0, IQR 2.6–3.6) than in the DS group (2.4, IQR 1.9–2.8) (*P* = 0.0054). However, baseline GH levels and adenoma volume were not different between these two groups (baseline GH levels: SA group, 40.9 *μ*g/L (IQR, 15.7–56.1 *μ*g/L) versus DS group, 26.4 *μ*g/L (IQR, 12.1–66.5 *μ*g/L), *P* = 0.22; adenoma size: SA group, 2110 mm^3^ (IQR, 1000–2500 mm^3^) versus DS group, 1470 mm^3^ (IQR 249–6160 mm^3^), *P* = 0.65).

As shown in Table 2, after 3 months of long-acting SSA therapy, the mean posttreatment GH level was 7.9 *μ*g/L (IQR, 2.9–34.6 *μ*g/L), and the mean percentage fall of GH was 69.6% (IQR, 32.4–90.5%). The mean posttreatment IGF-1 index was 2.0 (IQR, 1.3~2.5), while the mean percentage reduction of the IGF-1 index was 34.0% (IQR, 12.6–54.3%). The mean percentage reduction in tumor volume was 23.0% (IQR, 10.0–45.0%).

### 3.2. The Protein Expression Pattern of SSTR2 and SSTR5

**Figure 1:**
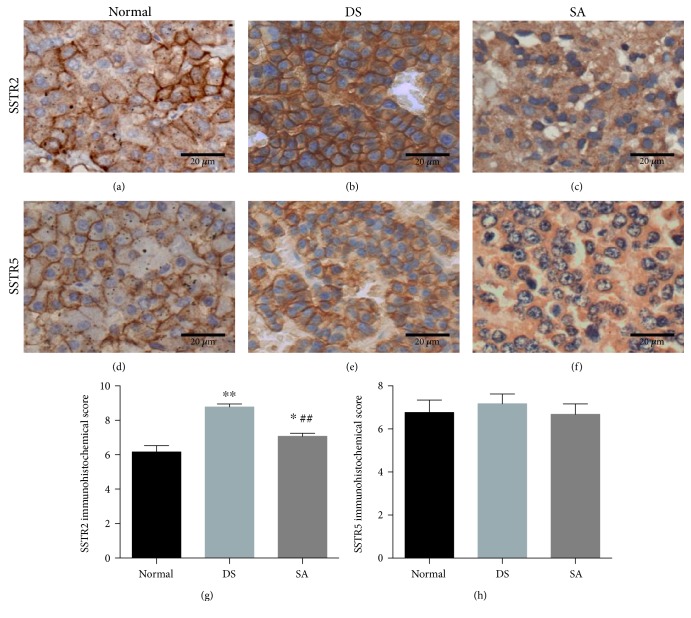
The protein expression patterns of SSTR2 and SSTR5: (a–c) protein expression pattern of SSTR2 in the normal pituitary group, direct surgery group, and SSA pretreatment group; (d–f) SSTR5 expression pattern; and (g and h) immunoreactive score of SSTR2 and SSTR5 for these three groups. Data are shown by mean ± SEM. ^∗^*P* < 0.05 and ^∗∗^*P* < 0.01 versus normal group; ^##^*P* < 0.01 versus DS group.

In order to detect the expression pattern of SSTR2 and SSTR5 in the normal pituitary and adenomas from both the DS group and SA group, we performed IHC analysis by using rabbit monoclonal antibodies against these two receptors. SSTR2 and SSTR5 from normal patients and the DS group exhibited predominantly membranous expression (Figures 1(a)–1(e)), while in the SA group, the expression of these two receptors was mainly localized in the cytoplasm (Figures 1(c) and 1(f)). Compared to the normal pituitary, the IRS of SSTR2 in the DS group was much higher (6 versus 9, IQR, 3.5–8.4 versus 7.2–10.3, resp., *P* < 0.01). However, pretreated adenomas (SA group) had a significantly lower score (8, IQR, 4.6–9.7) than those in the DS group (9, IQR, 7.2–10.3) (*P* < 0.01) (Figures 1(a)–1(c), 1(g)). Interestingly, there was no difference in the SSTR5 expression between the normal group (6, IQR, 5.0–8.6), DS group (6, IQR, 4.8–9.3), and SA group (6, IQR, 3.4–9.3) (*F* = 0.36, *P* = 0.69) (Figures 1(d)–1(f) and 1(h)).

### 3.3. Correlation between the Baseline Biomedical Characteristics of SA, DS, and the Entire Cohort and the Expression of SSTR2 and SSTR5

As Table 3 shows, in the SA group, there was a negative correlation between the SSTR2 score and the pretreatment GH levels (*r* = −0.312, *P* = 0.015), which suggested that a low SSTR2 score was associated with increased GH secretion. Moreover, this negative correlation was also found in the entire cohort (*r* = −0.228, *P* = 0.032), but not in the DS group.

The baseline IGF-1 index level was correlated with the SSTR2 score (*r* = 0.287, *P* = 0.043) in the SA group. There were no significant correlations between the SSTR2 score and the baseline tumor volume in any group. As for SSTR5, there were no significant correlations between it and the baseline biochemical indexes in these groups.

### 3.4. Correlation between the Clinical Biomedical Characteristics in SA Group and the Expression of SSTR2 and SSTR5

**Figure 2:**
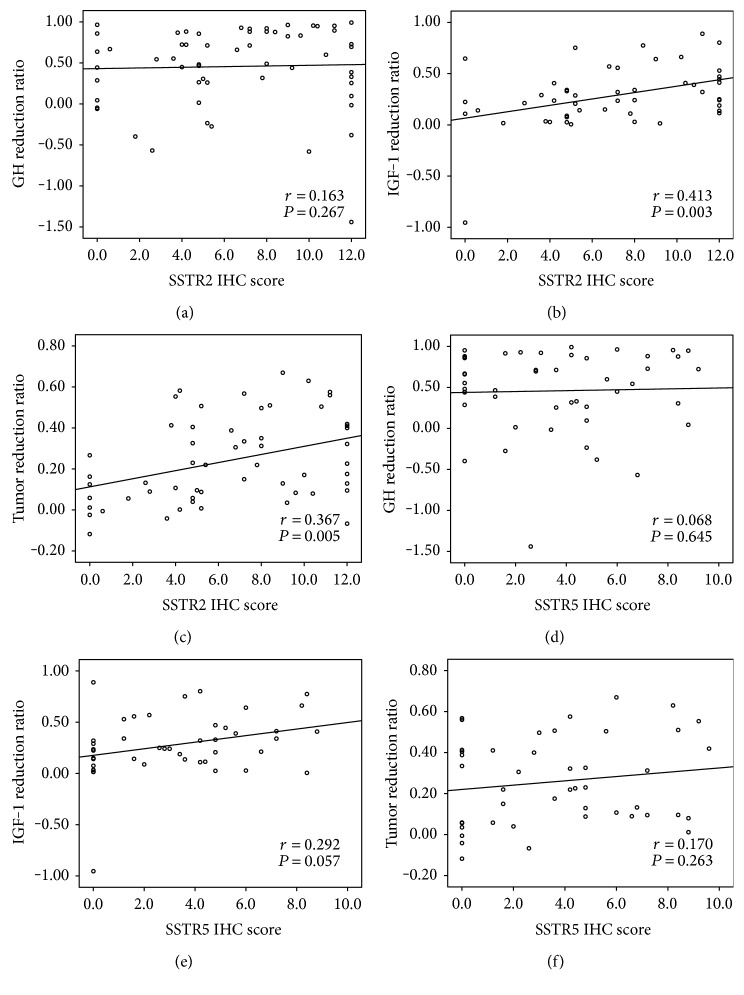
Correlation between GH (IGF-1 or tumor volume) reduction ratio and the expression of SSTR2 (or SSTR5): (a–c) correlation between the SSTR2 IRS score with GH reduction ratio, IGF-1 reduction ratio or tumor volume reduction ratio, respectively, and (d–f) correlation between SSTR5 IRS score with the same three indexes.

As shown in Table 4, there was a negative correlation between the SSTR2 score and post-SSA GH levels (*r* = −0.353, *P* = 0.006), although no significant correlation was found between the SSTR2 score and the GH reduction or the GH reduction percentage (Table 4 and Figure 2(a)). The relative IGF-1 index reduction (IGF-1 index reduction ratio) and the SSTR2 score were significantly positively correlated (*r* = 0.403, *P* = 0.004, Table 4; *r* = 0.413, *P* = 0.003, Figure 2(b), resp.), and there was a trend suggesting that the SSTR2 score was negatively correlated with the posttreatment IGF-1 index (*r* = −0.227, *P* = 0.094). Notably, the reduction of tumor volume was significantly correlated with the SSTR2 score (*r* = 0.367, *P* = 0.005, Figure 2(c)). These results suggested that the SSTR2 IHC score could predict the short-term biomedical response and tumor shrinkage with SSA treatment.

There were no significant correlations between the SSTR5 score and the clinical indexes, including baseline post-SSA GH, GH reduction, GH reduction ratio, IGF-1 index reduction, and tumor reduction (Table 4 and Figures 2(d)–2(f)). However, there was a trend suggesting that SSTR5 score was negatively correlated with posttreatment IGF-1 index (*r* = −0.244, *P* = 0.099), and the IHC score of SSTR5 tended to be positively correlated with the IGF-1 reduction percentage (*r* = 0.292, *P* = 0.057).

## 4. Discussion

In this study, we demonstrated for the first time in Chinese patients that the expression pattern of SSTR2 was different in the normal pituitary compared to somatotrophic adenomas. Moreover, SSTR2 expression was significantly lower in adenomas that were exposed to octreotide prior to surgery than those that did not receive pretreatment. We also determined that SSTR2 expression was correlated with the short-term biochemical and tumor volume response to SSA.

Somatostatin analogues (SSA) have been identified as the first line of medications in treating acromegalic patients [4, 19]. However, the response to SSA treatment is highly variable: some patients achieve full biochemical remission and a considerable reduction of tumor volume, whereas others are resistant (see Table 1 in Supplementary Material available online at https://doi.org/10.1155/2017/9606985) [13]. It is therefore urgent to find a way of identifying patients who may benefit from the SSA treatment.

Since SSTR2 and SSTR5 are the predominantly bound receptors for SSA, plenty of studies have focused on the predictive values of SSTR2 and SSTR5 expression. Results regarding the correlation between SSTR2 and SSTR5 mRNA and/or protein level and in vivo response to SSA have been subject to debate so far [7, 9, 14, 20]. Although there is a positive correlation between SSTR2 mRNA expression and the response of SSA, the mRNA level does not necessarily reflect the protein level or the functional activity due to translational and posttranslational regulation of protein synthesis [21]. Moreover, before the development of monoclonal antibodies against SSTR2 and SSTR5, IHC studies have mainly been limited to analyze the expression of only SSTR2 [22], partly because it is the most relevant receptor for SSA binding and the polyclonal antibodies against other SSTR subtypes are unsatisfactory [15].

Our results showed that the SSTR2 protein expression, but not the SSTR5, was much higher in acromegalic patients than normal subjects. This was supported by a study from Neto et al. [23] who reported that the mRNA expression of SSTR2 in somatotropinomas was higher than in the normal pituitary. Moreover, we also observed a significantly lower SSTR2 score after SSA treatment, whereas expression of SSTR5 remained unchanged. This is consistent with the previous reports from Casar-Borota, Fougner, and Plockinger [15, 22, 24].

The reason why SSA treatment could reduce SSTR2 IHC scores may be the receptor internalization of SSTR2. Basic research has demonstrated that SSTR2 could translocate from the cell membrane to the cytoplasm after SSA treatment [25–27]. Our IHC results also verified this phenomenon. In the normal pituitary and DS groups, the SSTR2 exhibited a predominantly membranous expression pattern. However, in the SA group, this receptor was more frequently localized in the cytoplasm. Taken together, these data suggested that SSA altered the expression and distribution pattern of SSTR2.

In summary, the IHC score of SSTR2 is a valuable indicator for the prediction of both biochemical and tumor size response to SSA and could be a clinically useful index to predict the short-term efficacy of SSA treatment in acromegalic patients. However, due to the small sample size of patients involved, further studies should be planned in order to investigate and validate our results. In this study, we performed the IHC test using monoclonal anti-SSTR2 and SSTR5 antibodies. Consistent with a previous study [15], SSTR2 expression, but not SSTR5, was a valuable indicator in predicting the short-term efficacy of SSA in acromegalic patients.

## Supplementary Material

Literature review about the predictive value of short- or long-term response to SSA treatment by the SSTRs expression. RT-PCR, IHC, and/or WB were used to detect the expression of SSTRs. SSTR, somatostatin receptor; RT-PCR, reverse transcription polymerase chain reaction; IHC, immunohistochemistry; WB, western blot; GH, growth hormone; IGF-1, insulin-like growth factor 1; OGTT, oral glucose tolerance test; SSA, somatostatin analogues.

## Figures and Tables

**Table 1:** Basic characteristics of the cohorts.

	Entire cohort	DS group	SA group	*P* value
Number (female/male)	93 (59/34)	30 (20/10)	63 (39/24)	0.82^a^
Age at diagnosis, y	41 ± 12	41 ± 13	43 ± 12	0.59^b^
GH, *μ*g/L	34.2 (13.5–57.9)	26.4 (12.1–66.5)	40.9 (15.7–56.1)	0.22^c^
IGF-1 index	2.9 (2.4–3.6)	2.4 (1.9–2.8)	3.0 (2.6–3.6)	<0.01^c^
Adenoma volume (*n* = 77), mm^3^	1900 (1000–2515)	1470 (249–6160) (*n* = 19)	2110 (1000–2500) (*n* = 58)	0.65^c^

DS, direct surgery group; SA, pretreatment with somatostatin analogues before surgery; GH, growth hormone; IGF-1, insulin-like growth factor 1. Data are mean ± SEM for age at diagnosis and median with interquartile range for GH, IGF-1 index, and adenoma volume. For adenoma volume, there are 77 patients available for analysis (for DS group, *n* = 19, while for SA group, *n* = 58). ^a^*Χ*^2^ test. ^b^Student's *t*-test. ^c^Student's *t*-test after log transformation.

**Table 2:** Clinical characteristics of the SA group before and after SSA treatment.

	SA group (*n* = 63)
Baseline GH, *μ*g/L	40.9 (15.7–56.1)
Post-SSA GH, *μ*g/L	7.9 (2.9–34.6)
% GH reduction	69.6 (32.4–90.5)
Baseline IGF-1 index	3.0 (2.6–3.6)
Post-SSA IGF-1 index	2.0 (1.3–2.5)
% IGF-1 index reduction	34.0 (12.6–54.3)
% tumor reduction	23 (10–45)

SA, pretreatment with somatostatin analogues before surgery; GH, growth hormone; IGF-1, insulin-like growth factor 1; SSA, somatostatin analogues. Data are median with interquartile range.

**Table 3:** Correlation between the baseline biomedical characteristics of SA, DS, and the entire cohort and the expression of SSTR2 and SSTR5.

	Baseline GH	Baseline IGF-1 index	Baseline tumor volume
SA group			
SSTR2	*r* = −0.312	*r* = 0.287	*r* = −0.079
	*P* = 0.015	*P* = 0.043	*P* = 0.561
SSTR5	*r* = 0.068	*r* = −0.001	*r* = −0.032
	*P* = 0.645	*P* = 0.997	*P* = 0.831

DS group			
SSTR2	*r* = −0.5	*r* = 0.099	*r* = −0.238
	*P* = 0.791	*P* = 0.61	*P* = 0.342
SSTR5	*r* = −0.159	*r* = 0.1	*r* = −0.195
	*P* = 0.402	*P* = 0.644	*P* = 0.342

Entire cohort			
SSTR2	*r* = −0.228	*r* = 0.019	*r* = −0.125
	*P* = 0.032	*P* = 0.866	*P* = 0.284
SSTR5	*r* = −0.029	*r* = −0.229	*r* = −0.108
	*P* = 0.8	*P* = 0.051	*P* = 0.391

SA, pretreatment with somatostatin analogues before surgery; DS, direct surgery group; GH, growth hormone; IGF-1, insulin-like growth factor 1; SSTR, somatostatin receptor. The Pearson correlation coefficient was used to analyze the correlations between the immunoreactive score (IRS) and clinical parameters.

**Table 4:** Correlation between the clinical biomedical characteristics in SA group and the expression of SSTR2 and SSTR5.

SA group	Post-SSA GH	GH reduction	% GH reduction	Post-SSA IGF-1 index	IGF-1 index reduction	% IGF-1 index reduction	% tumor reduction
SSTR2	*r* = −0.353	*r* = −0.14	*r* = 0.163	*r* = −0.227	*r* = 0.403	*r* = 0.413	*r* = 0.367
	*P* = 0.006	*P* = 0.342	*P* = 0.267	*P* = 0.094	*P* = 0.004	*P* = 0.003	*P* = 0.005
SSTR5	*r* = −0.044	*r* = 0.006	*r* = 0.068	*r* = −0.244	*r* = 0.222	*r* = 0.292	*r* = 0.17
	*P* = 0.765	*P* = 0.97	*P* = 0.678	*P* = 0.099	*P* = 0.153	*P* = 0.057	*P* = 0.263

SA, pretreatment with somatostatin analogues before surgery; GH, growth hormone; IGF-1, insulin-like growth factor 1; SSA, somatostatin analogues; SSTR, somatostatin receptor. The Pearson correlation coefficient was used to analyze the correlations between the immunoreactive score (IRS) and clinical parameters.
