# Supplementary material for: Expression of Somatostatin Receptor 2 in Somatotropinoma Correlated with the Short-Term Efficacy of Somatostatin Analogues
Source: Int J Endocrinol. 2017 Mar 15;2017:9606985. doi: 10.1155/2017/9606985 (PMC5370518; doi:10.1155/2017/9606985)
Supplement: Supplementary file 1 — Literature review about the predictive value of short- or long-term response to SSA treatment by the SSTRs expression. RT-PCR, IHC, and/or WB were used to detect the expression of SSTRs. SSTR, somatostatin receptor; RT-PCR, reverse transcription polymerase chain reaction; IHC, immunohistochemistry; WB, western blot; GH, growth hormone; IGF-1, insulin-like growth factor 1; OGTT, oral glucose tolerance test; SSA, somatostatin analogues. [file 9606985.f1.docx]

**Supplemental Table 1**. The predictive value of short- or long-term response to SSA by the SSTRs expression.

| Author & year | Study type | No | Type of SSTRs | Detection method | Antibody | Indexes | Conclusion |
| --- | --- | --- | --- | --- | --- | --- | --- |
| Taboada; 2008 | Retrospective | 22 | SSTR1-5 | RT-PCR | - | GH, IGF-1, tumor volume | SSTR2 mRNA level correlated positively with in vivo hormonal and tumor response to SSA. |
| Gatto, 2007 | Retrospective | 22 | SSTR2a, SSTR5 | IHC | Polyclonal | OGTT | SSTR2a, but not SSTR5, positive correlated with the percent suppression in the OGTT. |
| Olivera, 2013 | Prospective | 75 (65 was included in analysis) | SSTR1-5 | IHC | Monoclonal | GH and IGF-1 reduction | SSTR2a expression correlated with the response to octreotide and was reduced after octreotide treatment. |
| Gatto, 2015 | Retrospective | 36 | SSTR2a | IHC & RT-PCR | Monoclonal | GH and IGF-1 levels | SSTR2a IRS represents a valid tool in the clinical practice to identify acromegalic patients likely to be responders to adjuvant therapy with SSA |
| Fan, 2015 | Retrospective | 20 patients and 7 normal pituitary sample | SSTR2, SSTR5 | IHC | Monoclonal | GH | The expression of SSTR2 tended to be lower in the SSA non-responder group than in responders. |
| Wildemberg, 2013 | Retrospective | 88 (66 was included in analysis) | SSTR2a, DR2 | IHC | Polyclonal | GH and IGF-1 reduction | Low SSTR2A, but not DR2, expression is a negative predictive factor to response to SA. |
| Fougner, 2008 | Retrospective | 71 | SSTR2a | IHC and WB | Polyclonal | OGTT, IGF-1 | The clinical effect of octreotide correlates with the proportion of cells positive for SSTR2a in immunohistochemical staining, rather than the adenoma SSTR2a protein level. |
| Ferone, 2008 | Retrospective | 24 | SSTR2a, DR2, PRL | IHC | Polyclonal | GH and IGF-1 reduction | SSTR2a was positively correlated with in vitro and in vivo percent GH suppression by octreotide and with the chronic suppression of IGF-I by somatostatin analogs. |
| Gonzalez, 2014 | Retrospective | 60 | SSTR2, SSTR5 | IHC & RT-PCR | Polyclonal | GH and IGF-1 reduction | Neither SSTR2 or SSTR5 expression correlated with baseline or post-octreotide GH or IGF1 levels or tumor volume by either method. |
| Brzana, 2013 | Retrospective | 70 | SSTR2a | IHC | Polyclonal | GH, IGF-1, tumor volume | IHC SSTR2a positive status is a predictor of somatostatin receptor ligands response. |
| Casarini, 2009 | Retrospective | 39 | SSTR1-5 | IHC & RT-PCR | Polyclonal | GH, IGF-1, tumor volume | There was a positive correlation between the percentage of tumor reduction and SSTR1, SSTR2 and SSTR3 expression. |
